# Dr. Walker, on Vacciolation

**Published:** 1804-12-01

**Authors:** John Walker

**Affiliations:** Salisbury Square


### *To the Editors of the Medical and Physical Journal.*

GENTLEMEN,

THE estimable character on whom it has fallen to discover, and to reveal to a suffering world, the means of escaping the pestilential horrors of small-pox, was I think peculiarly happy in detecting the existence and the causes of *spurious* pustule.

On the subject of spurious pustule, &c. I offer you the following Address to the Governor of Minorca, written, as will appear from its contents, when I had not yet acquired that experience which I have since obtained.

"TO THE GOVERNOR FOX.

"John Walker, M. D. associated with Joseph Head Marshall, M. D. in an order from the Admiralty to take a passage on board of the Endymion, for the purpose of bringing to this part of the world the new discovery of the cow-pox inoculation, thinks it his duty to return thanks, on the part of himself and colleague, to the Governor at Minorca, for his liberal attention to themselves personally as well as to their mission, and to inform him of their proceedings.

"When the native magistrates, the Jurats, had given them an audience, at the instance of the Governor, and expressed their approbation* of the new practice, they immediately proceeded to inoculate the troops and seamen of the Navy, &c.

"Other men and children attached to the British service have been inoculated, &c.

"Candour obliges us to confess, that the disease has been produced in but a very partial way. The effect upon the system has not been so great as we have witnessed it in England; it passed away in about half the time. In many instances, it has not been produced at all.

"Information of this seemed due to the natives. Their safety might be concerned in it. The honour of the medical profession, as well as individual character, should be supported: and it ought not to appear to them, that the British government had in any way patronized a species of charla-

* Without this the Governor told us he could not, by treaty, consent to the inoculation being introduced into the island.

charlatanerie in place of a practice important to humanity, and which, originating in England, is now spreading itself rapidly to different parts of the world.

"The explanation of the cause of the apparent degeneration of the disease, or of the production of a new one, was attempted; and they were fully satisfied. They were informed that threads impregnated with vaccine virus were brought from villages in Gloucestershire, and matter, still more recent, from London; that the latter was used successfully in the Endymion; that hence fresh virus was obtained, which effectually produced the disease at Gibraltar; that the disease was also produced in the Florentino, on her way from Gibraltar to this island; but that here we in vain attempted to excite the disease by the virus brought from England, as well as by that more recently collected on the way; that we then brought a boy from on board the Florentino, and inoculated the children at the Foundling Hospital with matter taken from his arm, and that this produced the diminished effect already mentioned; that on our witnessing this new appearance of the disease, we were alarmed, and concluded, that one of us should return to the Rock, to obtain fresh matter, as the medical men there had undertaken to keep up the disease as well as to preserve the matter of it; or, in case of failure there, to proceed to England; that the other should continue to attempt to excite with the yet untried threads of virus; that this attempt has been abortive, and that nothing but the diminished or degenerated disease had been propagated in this island. Time may fully determine whether this slight affection will at all fortify the system against the small-pox. My opinion is, that it may moderate the effects of it, although I fear that it will not entirely prevent it.

"As the confluent small-pox has lately been raging in the island, I thought it right to recommend it, remarking, that it might be, in comparison of the fully marked cow-pox, what one coat of lime would be to a wall which they wished to thoroughly clean and whiten (pointing to a clouded or stained part of their town-hall) would be to a full proper whitening.

"But whence arose the failure of the virus to which we suppose the present disappointment referable? On packing up that which we obtained since our leaving England, it happened that in sealing it up a degree of heat was applied, which at the time excited some fear that the matter might be injured. The fear seems to have been realized. As to the failure of the virus brought from England, we seem obliged obliged to refer it less to its age than to some incautious exposure to the air.

"But why was the virus taken from the sailor-boy ineffectual to produce a disease bearing the character of the undoubtedly genuine cow-pox? The lad was a Spaniard; we did not understand each other's language. First, Perhaps he had had the small-pox. Secondly, As we only tryed him through failure of what we had depended upon, perhaps we, under fear of utterly failing, had taken the matter from his arm at rather too late a period, when the limpid virus had passed to a state of pus. Or, thirdly, He might have some other disease upon him. It is a fact that there was at last discovered on him a swelling with considerable discharge, and this perhaps prevented him from receiving the disease in its full force, and the matter of consequence, taken from him, from giving it."

*Aboard of the Alexander, Mahon Harbour,* 19, *x*, 1800.

As I had hitherto only practised the Jennerian inoculation in the vale of Gloucestershire, which had given it birth, the spurious pustules which arose in Minorca were the first I had seen. I however yet believe, that unequivocally spurious, or degenerated, cow-pock, produces all the effects which have lately been attributed to the true; viz. a kind of partial and temporary protection against the violence of small-pox; and with this persuasion on my mind, I can only refer every *failure* to the use of improper matter.

I informed the Jurats, that on our recovering the matter, my colleague or myself would return to the isle, or that we would send it to the surgeon-general, who had accompanied me to the hall, and who promised to introduce it. On Marshall's rejoining me at Malta, he informed me, that immediately on his return to Minorca from Gibraltar, he re-inoculated an officer's child with full effect; that he afterwards submitted it to variolous inoculation without effect, and that the natives assured him, that while several had died there of the small-pox, even under inoculation, those who had had the imperfect cow-pock had been very little affected with the small-pox.

I suppose we shall shortly have the Report of the very respectable association on the case at Fulwood's Rents. Will they find it to have been varicella, wasser pocken, diminished small-pox after spurious cow-pock, or what? In the bills of mortality for August 21, October 2, and 16, I find three deaths attributed to chicken-pox. If these eruptions, whatever they were, had been after vacciolation or or vaccævaration, they must have been fatal cases of small-pox after cow-pock.

Lately called on to see a case of variola after vacciola, I found a child covered with small-pox, and immediately declared it to be such. On examining its arms, and not finding any cicatrix, I informed the parents that it had never had the cow-pock. Another child was shown to me, on whom there was distinctly characterised cicatrix; I told them that this afforded so strong a presumption that it was fully protected, that I would recommend its sleeping with the small-pox patient. They have slept together from the beginning, said the father and mother. Some weeks have now elapsed, the child's protection stands confirmed; and the mother now recollects, that the Doctor took a great deal of matter from its arm, but none from the one which has taken the small-pox.

A father called on me a few days ago, and told me, that of two children which I had inoculated last spring, one was now covered with small-pox, the other sickening; and that he was advised to advertise it. On consulting the Register, I found them both marked perfect cases; and told him, it was impossible for either of them to be infected with small-pox. I immediately called on our Vice President, John Ring, and challenged him to come and detect my failure, remarking, that I could scarcely expect my own report, if favourable, to be confidently received. He had the goodness to accompany me, and on our seeing the child, he immediately declared it chicken-pox.

The applications for matter at the Central House, are more numerous than ever; there is not yet any falling off in the inoculations.

Your's, respectfully,

*Salisbury Square*, 15 *xj*, 1804. JOHN WALKER.

